# Could Questions on Activities of Daily Living Estimate Grip Strength of Older Adults Living Independently in the Community?

**DOI:** 10.1155/2012/427109

**Published:** 2012-03-18

**Authors:** Jessica Simard, Maude Chalifoux, Véronique Fortin, Maude Jeanson Archambault, Anne St-Cerny-Gosselin, Johanne Desrosiers

**Affiliations:** ^1^Health and Social Services Centre, University Institute of Geriatrics of Sherbrooke, Sherbrooke, QC, Canada J1H 4C4; ^2^School of Rehabilitation, Faculty of Medicine and Health Sciences, Université de Sherbrooke, Sherbrooke, QC, Canada J1H 5N4; ^3^Centre de Réadaptation Estrie, Sherbrooke, QC, Canada J1G 1B1; ^4^Faculty of Medicine and Health Sciences, Université de Sherbrooke, Sherbrooke, QC, Canada J1H 5N4; ^5^Research Centre on Aging, Health and Social Services Centre, University Institute of Geriatrics of Sherbrooke, Sherbrooke, QC, Canada

## Abstract

The aim of this study was to identify questions that could estimate grip strength. Twenty-six questions about the degree of perceived difficulty performing manual tasks as well as two questions concerning self-rated grip strength were developed and completed by 123 community-dwelling older adults, followed by grip strength measurements using a Martin vigorimeter. Multiple regression analyses with all of the participants revealed that the question about the difficulty of opening a jar (question 4) was most associated with grip strength. When analyses were done by gender, the same question showed the best correlation for women, whereas the one for men was self-rated grip strength compared with people the same age (question 28). For the women, age and question 4 together explained 54% of the variance in their grip strength and for the men, age and question 28 explained 46%. Further studies are needed to identify other information that could help to better estimate grip strength for use in epidemiological surveys.

## 1. Introduction

Many older adults have to deal with disabilities in mobility, activities of daily living (ADL), or instrumental ADL [1]. Among other impairments, decrease in grip strength may be related to these aging-related disabilities. Grip strength is often used as an indicator of general muscle strength and is a good estimate of upper limb strength [2]. It is an easily measurable variable [3, 4] and a good indicator of frailty and future disability [5–7]. Sufficient grip strength is necessary to perform many ADLs and may be indicative of functional independence [8].

Using a dynamometer is the best way to measure grip strength. No other valid objective method is currently available. Therefore, grip strength measurements cannot be included in large epidemiological studies, which are frequently conducted by phone or mail questionnaires. These questionnaires present many advantages when compared to face-to-face interviews. They are cheaper, they do not require specialized equipment, they take less time, and they overcome difficulties related to population geography. Therefore, the development of an alternative to direct measurement of grip strength could allow an estimated measure to be included in population-based surveys on aging and activity limitations. Adding grip strength estimation to such surveys could help to appraise the vulnerability of the older population with the aim of promoting health and implementing preventive strategies as well as organizing and planning services to address these population's needs [9].

The objective of the study was to identify questions about tasks or daily activities done with the hands that could be good estimators of objectively determined grip strength.

## 2. Material and Methods

### 2.1. Development of a Hand Activity Questionnaire

A two-section questionnaire comprising 28 questions was developed by the authors (see the Appendix). The first section contains 26 questions about the degree of difficulty (0: none at all; 1: a little; 2: moderate; 3: a lot; do not know/not applicable) in performing different tasks of daily living involving grip strength, such as opening a jar, carrying a bag of groceries, and holding a dictionary. The choice of these tasks was first based on an analysis of tasks or activities that require different amounts of grip strength. Then the relevance of these activities was discussed with experienced occupational therapists and physical therapists. Modifications were made, which led to the current version. The second section of the questionnaire contains two questions about self-rated grip strength as compared to (1) the participants' own strength 10 years ago (0: similar, 1: slightly reduced, 2: moderately reduced, 3: considerably reduced), and (2) other people at the same age (0: very good, 1: good, 2: fair, and 3: poor).

### 2.2. Empirical Data Collection

In order to identify questions that could be the best estimators of grip strength, a cross-sectional study was carried out with people aged 50 years and older living independently in the community (home or private residence for older adults) in the province of Quebec, Canada, and presenting different levels of activity and abilities. People suffering from acute pain as well as those who had moderate-to-severe cognitive problems (based on clinical judgement) were excluded from the study. All participants signed an informed consent form. The study was approved by the research ethics board of the Health and Social Services Center, University Institute of Geriatrics of Sherbrooke.

To be representative of the population, participants were recruited as much as possible considering demographics for age and gender using the 2008 age pyramid for Quebec [10]. Using convenience and snowball samplings, recruitment was carried out in different environments frequented by older adults (home, private residence for older adults, mall, and leisure center) and among the researchers' family circles.

Participants were evaluated once by the first five authors in a 30-minute face-to-face interview at the location where they were recruited, usually their own living environment. They were asked to complete a sociodemographic questionnaire followed by the hand activity questionnaire described earlier. Then, the grip strength of both their hands was measured using a Martin vigorimeter (Gebrüder Martin, Tuttlingen, Germany) [11]. This instrument consists of a rubber bulb which is grasped by the hand, a rubber connecting tube, and a manometer. The pressure in the bulb is registered on the manometer. Three sizes of bulbs are available. The Martin vigorimeter was chosen over the Jamar dynamometer since it is more suitable for an aging population as it does not put pressure on weak or painful joints [12]. In addition, these two instruments are highly correlated (0.90 and 0.89, according to the hand) [12]. In the present study, two vigorimeters were used, one with the pressure measured in kPa and the other in pounds per square inch. To be consistent, the kPa data were transformed into lbs/inch^2^ (1 lb/inch^2^ = 6.9 kPa) [13]. Grip strength was measured following the procedure recommended by Mathiowetz and colleagues [14]. The subjects were seated with their shoulder adducted and neutrally rotated, elbow flexed at 90°, forearm in neutral position, and wrist between 0° and 30° of extension. Three measures of each hand were taken alternately, starting with the dominant hand and using the largest bulb for all subjects. The average of the three measures of the dominant hand was used for analyses. Each interviewer participated in a training and standardization session before starting data collection.

### 2.3. Statistical Analyses

Based on an *α* significance level of 5% and power of 80%, a sample of 123 subjects was required to be able to detect a correlation of 0.25 or over as statistically significant (bilateral test) [15].

Pearson's correlation coefficients were first calculated between each question on the hand activity questionnaire (independent variables) and the average grip strength of the dominant hand, first for all the participants and then separately for the women and men. Secondly, independent variables including sociodemographic variables that were significantly (*P* < 0.05) correlated with grip strength were put into a model. The data normality of the dependent variable (grip strength) was verified visually with histograms and statistically with the Kolmogorov-Smirnov test. Multiple regression analyses were carried out with all possible models [16] in order to choose the best final model using a stepwise approach. A residual analysis was done to verify the basic assumptions. SPSS 17 and SAS 9.2 applications were used for the statistical analyses.

## 3. Results

As required, a total of 123 community-dwelling adults (79 women; 64%) aged 50 years and older participated in the study. Most of the participants were right-handed, retired, and considered themselves to be active (see Table 1). As expected, the men were stronger than the women (12.8 ± 3.6 lbs/inch^2^ versus 7.9 ± 2.7 lbs/inch^2^).

Pearson's correlation coefficients showed that, in general, questions statistically correlated with objective grip strength were similar for the women and men (Table 2) even if the magnitude of correlations may have differed. Two questions, (20: opening a bottle and 17: wringing out a towel), were found to be correlated with grip strength for the women only (and not for the men), whereas question 27 (self-rated strength compared to 10 years ago) was correlated only to the men (and not to the women). Two other questions, (19 and 25), were associated with grip strength for all the participants but were no longer significant when analyses were done by gender. 

Multiple regression analyses carried out with all the participants showed that question 4 (“*Do you have difficulty opening a jar of jam (or jar of something else) that has never been opened*?”) was the one most associated with grip strength. When analyses were done by gender, the same question was the best correlation to the women, while for the men the question most related to grip strength was 28 (“*How would you rate your grip strength compared to other people in your age*?”) (Table 3). In addition to these questions, age was the variable best correlated with grip strength. For the women, age and question 4 together explained 54% of the variance in their grip strength, while for the men, age and question 28 explained 46% of the variance (Table 3). The women reported having differing degrees of difficulty when answering question 4 (none at all: 10 (12.7%); a little: 32 (40.5%); moderate: 15 (19.0%); a lot: 21 (26.6%); one did not know). The distribution of the answers to question 28 was more homogenous since almost all of the male participants rated themselves as having “very good” [15 (34.1%)] or “good” [27 (61.4%)] grip strength compared to other people their age. Only 2 men (4.5%) but 13 (16.5%) women rated their strength as fairly good. In addition, more female (82.3%) than male participants (64.5%) rated their grip strength as being slightly, moderately, or substantially reduced compared to 10 years ago, with the others estimating their strength as similar. 

## 4. Discussion

The aim of this study was to identify questions about tasks or daily activities done with the hands that could estimate objective grip strength for potential use in phone interviews or mail questionnaires. Twenty-eight questions were first developed and then correlated with the dominant hand's grip strength measurement. As expected, many questions were found to be correlated with grip strength. Although the magnitude of the correlation coefficients differed by gender; similar questions, with some exceptions, were identified as being associated with grip strength. However, regression analyses identified a different question for the men (number 28) and women (number 4) as being the best correlates with grip strength. For the men, the question concerned self-rated strength, whereas the one for women related to the degree of difficulty in performing a daily task. This difference might, at least in part, be attributable to the type of tasks covered in the questionnaire. These tasks might not be difficult enough to require a high degree of strength from men. Since they are stronger than women and begin to lose their muscle strength later in life [5, 17], men report less difficulties than women in many tasks. Our results support this later decline in grip strength of the men as a higher percentage of them rated their grip strength as similar to 10 years ago than the women, and more of them rated it as “very good” or “good.” Including heavier tasks in the questionnaire of hand activities might have revealed stronger correlations with grip strength among the men. Interestingly, the most relevant question for the men concerned self-rated grip strength. This question was selected for the questionnaire, in addition to difficulty doing tasks, based on the frequent question regarding self-rated health extracted from the OARS Multidimensional Functional Assessment Questionnaire (OMFAQ) [18], a question that has been recognized as a reliable indicator of objective health status and mortality [19]. 

Among the sociodemographic data, age was found to be closely related to grip strength, which is well known [5, 20, 21]. Together, those variables were able to explain only a part of the variance in grip strength (54% and 46% for women and men, resp.). An estimate will never replace direct measurement of grip strength, which is influenced by various factors such as morbidity and nutritional status [22], socioeconomic and financial circumstances in old age [23], lifestyle, and culture [24] as well as anthropometric variables [21, 25]. Therefore, other variables should be considered to optimize grip strength estimation for use in phone interviews or mail questionnaires. However, the challenge is to find valid yet simple methods to evaluate those variables. 

This study has some limitations. First, our eligibility criteria were chosen to include people aged 50 years and older. However, the reduction in grip strength is accentuated after age 75 [26], and many participants had good grip strength since they were mostly younger. Second, participants were not randomly selected and may not be representative of the population of older adults living independently in the community. However, they were quite representative in terms of age and gender. Also, the sample size was first calculated without considering gender. Since analyses had to be done separately for the men and women, the smaller proportion of men in the present sample reduces the power to detect significant correlations. 

To our knowledge, this is the first published study aimed at estimating grip strength using the kind of questions that could be asked over the phone or in mail questionnaires. Considering the clinical relevance of grip strength [7, 26], inclusion of an estimated measure in population-based surveys on aging and activity limitations could help to characterize both health and functional status of the senior population. In addition, this type of information might be helpful in identifying older people who are frail or at risk of becoming so, in order to promote the functional independence of older adults and thus improve their quality of life [27]. Future research should include more participants, particularly men, in order to enhance the external validity of the study. Also, future studies should include older participants (65 years and older) since epidemiological studies on activity limitations are particularly relevant for this population. Finally, using older participants with poorer grip strength might reveal stronger correlations with difficulty doing manual tasks. 

## 5. Conclusions

The purpose of this study was to identify questions, mainly related to tasks or daily activities done with the hands, which would be the best estimators of grip strength. From a questionnaire composed of 28 questions, only one question per gender was found to be the best estimator of grip strength, and only a small proportion of the variance in grip strength was explained by these questions. This proportion increases when age is considered. Further studies are needed to identify other variables that could help to better estimate grip strength for use in large epidemiological surveys. 

## Figures and Tables

**Table 1:** Characteristics of the participants.

		All *n* = 123	Women *n* = 79	Men *n* = 44
Age (mean, SD)		66.1 (12.6)	67.9 (13.1)	62.8 (10.9)
		Freq (%)	Freq (%)	Freq (%)

Hand dominance	Right	108 (87.8)	72 (91.1)	36 (81.8)

Activity level	Very active	27 (22.2)	17 (21.5)	10 (22.7)
	Active	73 (59.3)	50 (63.3)	23 (52.3)
	Inactive	22 (17.9)	12 (15.2)	10 (22.7)
	Sedentary	1 (0.8)	0 (0.0)	1 (2.3)

Occupation	Work	45 (36.6)	24 (30.4)	21 (47.7)
	Retirement	62 (50.4)	41 (51.9)	21 (47.7)
	Voluntary work	15 (12.2)	13 (16.5)	2 (4.5)
	Other	1 (0.8)	1 (1.3)	0 (0.0)

**Table 2:** Significant Pearson's correlation coefficients between questions about difficulty doing tasks and grip strength.

Tasks	All participants		Women		Men	
	*n* = 123		*n* = 79		*n* = 44	
	*r*	*P* value	*r*	*P* value	*r*	*P* value
Q4. Open jam jar	−0.606	<0.001	−0.486	<0.001	−0.363	0.015
Q3. Carry grocery bag	−0.480	<0.001	−0.379	0.001	−0.388	0.01
Q20. Open bottle	−0.431	<0.001	−0.328	0.003		ns
Q12. Carry pot of water	−0.427	<0.001	−0.360	0.001	−0.423	0.005
Q7. Hold dictionary	−0.385	<0.001	−0.315	0.007	−0.427	0.005
Q17. Wring out towel	−0.367	<0.001	−0.263	0.022		ns
Q28. Self-rated strength compared to others	−0.361	<0.001	−0.425	<0.001	−0.323	0.03
Q26. Open bag of chips	−0.360	<0.001	−0.350	0.002	−0.367	0.01
Q27. Self-rated strength compared to 10 years ago	−0.344	<0.001		ns	−0.376	0.01
Q2. Carry 2-liter container	−0.322	<0.001	−0.258	0.02	−0.351	0.02
Q21. Pour water from jug	−0.321	<0.001	−0.288	0.01	−0.351	0.02
Q19. Stir a thick mixture	−0.315	<0.001		ns		ns
Q25. Open 2-liter carton	−0.266	0.004		ns		ns

**Table 3:** Variables most associated with grip strength.

	Independent variables	Regression coefficients	Cumulative *R* ^2^	*P* values
Women *n* = 79	Age	−0.13		0.0043
	Question 4	−0.66		<0.001
	Intercept	17.61	0.54	<0.001

Men *n* = 44	Age	−0.20		0.0095
	Question 28	−1.97		<0.001
	Intercept	26.9	0.46	<0.001

Question  4: do you have difficulty in opening a jar of jam (or jar of something else) that has never been opened?

Question  28: how would you rate your grip strength compared to other people in your age?

## Appendix

### Hand Activity Questionnaire

Section 1

1. Do you have difficulty in pressing a half-empty tube of toothpaste?
2. Do you have difficulty in picking up 2 liters of milk/juice from the fridge and carrying it to the table?
3. Do you have difficulty in holding and carrying a heavy bag of groceries (bag with handles) over a short distance?
4. Do you have difficulty in opening a jar of jam (or jar of something else) that has never been opened?
5. Do you have difficulty in turning a round door knob?
6. Do you have difficulty in holding a telephone handset?
7. Do you have difficulty in holding a dictionary/telephone directory with one hand?
8. Do you have difficulty in using a cleaning product with a trigger spray (like Windex)?
9. Do you have difficulty in turning on a round tap that is tightly turned off?
10. Do you have difficulty in holding a hammer and hammering a nail?
11. Do you have difficulty in cutting a steak with a knife?
12. Do you have difficulty in carrying a medium-sized pot half full of water (e.g., to cook pasta)?
13. Do you have difficulty in holding a hair dryer?
14. Do you have difficulty in holding a glass when filling it under the tap?
15. Do you have difficulty in carrying a plate full of food?
16. Do you have difficulty in ironing your clothes?
17. Do you have difficulty in wringing out a towel to remove all the water?
18. Do you have difficulty in cutting things up?
19. Do you have difficulty in stirring a thick mixture with one hand (e.g., Rice Krispies with marshmallows, fudge)?
20. Do you have difficulty in opening a bottle of water or soft drink that has never been opened?
21. Do you have difficulty in pouring water from a full kettle or jug?
22. Do you have difficulty in opening a can with a manual can opener?
23. Do you have difficulty in pressing a half-full shampoo bottle?
24. Do you have difficulty in unlocking a door with a key?
25. Do you have difficulty in opening a new 2-liter carton of milk?
26. Do you have difficulty in opening a bag of chips or the wrapping on a granola bar that has never been opened?

Section 2

(27) How would you rate your current grip strength compared to the way it was 10 years ago?
(28) How would you rate your grip strength compared to other people in your age?
